# MicroRNAs That Contribute to Coordinating the Immune Response in *Drosophila melanogaster*

**DOI:** 10.1534/genetics.116.196584

**Published:** 2017-07-13

**Authors:** Magda L. Atilano, Marcus Glittenberg, Annabel Monteiro, Richard R. Copley, Petros Ligoxygakis

**Affiliations:** * Laboratory of Cell Biology, Development and Genetics, Department of Biochemistry, University of Oxford, OX1 3QU, United Kingdom; † Sorbonne Universités, Université Pierre et Marie Curie University Paris 06, Centre Nationnal de la Recherche Scientifique, Laboratoire de Biologie du Développement de Villefranche-sur-mer, 06230, France

**Keywords:** innate immunity, immune response regulation, miRNAs, *Drosophila melanogaster*, host–pathogen interactions, Genetics of Immunity

## Abstract

Small noncoding RNAs called microRNAs (miRNAs) have emerged as post-transcriptional regulators of gene expression related to host defenses. Here, we have used *Drosophila melanogaster* to explore the contribution of individual or clusters of miRNAs in countering systemic *Candida albicans* infection. From a total of 72 tested, we identify 6 miRNA allelic mutant backgrounds that modulate the survival response to infection and the ability to control pathogen number. These mutants also exhibit dysregulation of the Toll pathway target transcripts *Drosomycin* (*Drs*) and *Immune-Induced Molecule 1* (*IM1*). These are characteristics of defects in Toll signaling, and consistent with this, we demonstrate dependency for one of the miRNA mutants on the NF-κΒ homolog Dif. We also quantify changes in the miRNA expression profile over time in response to three pathogen types, and identify 13 mature miRNA forms affected by pathogens that stimulate Toll signaling. To complement this, we provide a genome-wide map of potential NF-κB sites in proximity to miRNA genes. Finally, we demonstrate that systemic *C. albicans* infection contributes to a reduction in the total amount of branch-chained amino acids, which is miRNA-regulated. Overall, our data reveal a new layer of miRNA complexity regulating the fly response to systemic fungal infection.

## Abstract (precis)

Atilano *et al.* present a *Drosophila* post-infection survival screen that takes advantage of a library of miRNA mutant flies. Using genome wide microarray..

*DROSOPHILA melanogaster* has led to some significant discoveries regarding the innate immune pathways involved in countering a wide range of microbial infections [reviewed in Kounatidis and Ligoxygakis (2012) and Bouchon *et al.* (2014)]. Microbial detection by pattern recognition receptors (PRRs) or sensors of “danger signals” leads to the activation of signaling cascades that result in expression of immune effectors and regulators.

The Toll and Immune Deficiency (IMD) signaling pathways regulate the activation of the nuclear factor κB (NF-κB) transcription factor homologs Dif/Dorsal and Relish, respectively [reviewed in Kounatidis and Ligoxygakis (2012) and Bouchon *et al.* (2014)]. Following Toll activation, a receptor–adaptor complex, which includes the death domain-containing proteins dMyD88, Pelle, and Tube, transmits the signal to the IκB homolog Cactus. The latter is then targeted for degradation, which leaves NF-κB proteins Dif/Dorsal free to translocate to the nucleus and regulate hundreds of genes (De Gregorio *et al.* 2001). Among others, Toll signaling triggers the transcription of several effector molecules including antimicrobial peptide (AMP) genes, such as *Drosomycin* (*Drs*). Dif enters the nucleus 20 min after infection (Ligoxygakis *et al.* 2002). Approximately 40 min following Dif nuclear entry, expression of the Toll pathway PRR (and target of Dif), PGRP-SA (peptidoglycan recognition protein-SA) is 10 times that of the basal level (De Gregorio *et al.* 2001, 2002). However, intracellular homeostasis of the pathway remains relatively unexplored. This is especially pertinent in the light of recent data that indicate that upon Toll induction, Pelle phosphorylates Cka of the STRIPAK (Striatin-interacting phosphatase and kinase) complex thus releasing Hippo signaling, which in turn prevents *cactus* transcription leading to the sustained activation of the Toll pathway (Liu *et al.* 2016).

MicroRNAs (miRNAs) are small noncoding RNAs, which act as post-transcriptional regulators of gene expression. MiRNAs are initially transcribed in the nucleus as a primary miRNA transcript by polymerase II, which are then cleaved into precursor miRNAs (pre-miRNAs) by the protein complex made up by nuclear RNase III protein Drosha and the double-stranded RNA-binding protein Pasha. The pre-miRNAs are then exported to the cytoplasm by the protein Exportin 5, where they are further processed by RNase III enzyme Dicer-1 with the help of double-stranded RNA-binding protein Loquacious to produce ∼22 nucleotide-long miRNA–miRNA* duplexes. Finally, one of the miRNA strands is loaded into the RNA-induced silencing complex (Risc), containing Argonaut-1 (Ago-1), for targeting of gene expression (Ha and Kim 2014).

Approximately 60% of all protein-coding genes have been estimated to be under miRNA control (Grun *et al.* 2005). A given miRNA can regulate several target transcripts, while an mRNA transcript might be targeted by multiple miRNAs. These RNAs play crucial roles in key biological processes, such as cell proliferation, cell fate, differentiation, apoptosis, and survival (Cayirlioglu *et al.* 2008; Bejarano *et al.* 2010; Weng and Cohen 2012; Morante *et al.* 2013; Chen *et al.* 2014). miRNAs have also emerged as important regulators of the host innate immune response and host–pathogen-interactions. Garbuzov and Tatar revealed that in *Drosophila*, 20-HE may regulate expression of the antimicrobial peptide Dpt via let-7 miRNA by interacting with the 3′-UTR of the target gene and repressing its translation (Garbuzov and Tatar 2010). These authors proposed that let-7, induced at the same time as *Dpt* by 20-HE, may set the limit on expression levels of *Dpt* by negatively regulating it to set a threshold for the AMP following immune induction, thus avoiding overstimulation of the innate immune system. Two other studies in *Drosophila* have indicated that miRNA-8 plays a role in maintaining the innate immune response (Choi and Hyun 2012; Lee and Hyun 2014). In these studies, miRNA-8 has been shown to regulate the levels of basal expression of AMPs such as *Drs* and *Dpt*. They revealed that, in the absence of infection, miRNA-8 targets the Toll receptor and Dorsal. By targeting these proteins, miRNA-8 ensures that, when the immune system is not stimulated, the levels of AMPs such as *Drs* are kept low. The miRNA cluster 959–964 has also been associated with immune response regulation in *Drosophila* (Vodala *et al.* 2012). In this case, the miRNA 959–964 cluster mutants revealed altered levels of mRNAs involved in immune responses, including *Drs*.

In addition, an *in silico* study by Fullaondo and Lee (2012) predicted that several other miRNAs may be involved in regulating the innate immune system, although, as yet, their predictions have not been tested due to the lack of available mutants. In fact, little is known about the *in vivo* role of miRNAs in innate immunity across species. Recently, the construction of a large collection of *D. melanogaster* miRNA allelic mutants was reported (Chen *et al.* 2014). Here, we utilize this collection to show that individual or clusters of miRNAs alter the ability of flies to cope with systemic infection, impacting on survival, the control of pathogen number, and the production of Toll-dependent transcripts.

## Materials and Methods

### Fly stocks

Flies obtained from the Bloomington *Drosophila* Stock Centre: c564 > Gal4 driver (#6982); UAS-myr_mRFP (#7118); *Dif^1^* loss-of-function allele (#36559); UAS-miR-277^Sponge^ (#61408); the control backgrounds *w^1118^* (#5905), *y^1^w^1^* (#1495), and the isogenic wild-type strain (#25174; originally from the *Drosophila* Genetics Reference Panel); and the collection of miRNA allelic mutants (Chen *et al.* 2014). Fly lines carrying UAS (upstream activating sequence)-RNA interference (RNAi) constructs were obtained from the Vienna *Drosophila* RNAi Centre: PGAP5^RNAi^ (#47953); Exp5^RNAi^ (#31706 and #31707); Dcr-1^RNAi^ (#24667); Loqs^RNAi^ (#22453); and Myd88^RNAi^ (#25402). Yolk > Gal4 was kindly provided by Bruno Lemaitre. We also used the previously described strains UAS-Ago-1 and UAS-Dcr-1 (Dietzl *et al.* 2007), and *miR-124^Δ6^* (Sun *et al.* 2012). All flies were raised on maize malt molasses food in a light-dark (12-hr cycle) incubator at 25° and 60% humidity. Flies were shifted to 30° and 60% humidity following infection for microarray, branch-chained amino acid (BCAA) quantification experiments, and those involving miRNA allelic mutants. Flies were shifted to 30° and 60% humidity 2 days prior to and then during infection for UAS-RNAi/UAS-miR-277^Sponge^/UAS-protein overexpression experiments.

### Pathogens and preparation

*Candida albicans* strain SC5314 was streaked intermittently from a −80° stock onto Sabouraud Agar (SGA), grown at 30° for 36 hr, and stored at 4° for up to a week. The inoculant was prepared from a single colony grown in 10 ml Sabouraud Broth (SGB) for 18–21 hr at 30° and shaken at 200 rpm; this was washed and resuspended in PBS to an OD_600_ of 0.95–1.05 (readings taken with a NanoDrop 1000 spectrophotometer; Thermo Fisher Scientific) and further diluted fourfold in PBS for injection into flies. *Staphylococcus aureus* strain NCTC-8325 was streaked intermittently from a −80° stock onto Tryptic Soy Agar, grown at 30° for 36 hr, and stored at 4° for up to a week. The inoculant was prepared from a single colony grown in 10 ml Tryptic Soy Broth for 16–18 hr at 30° and shaken at 200 rpm; this was washed and resuspended in PBS to an OD_600_ of 0.36 and further diluted 1000-fold in PBS for injection into flies. *Escherichia coli* strain 1106 was cultured in a manner similar to *S. aureus*, but Luria-Bertani agar/broth was used and the OD_600_ 0.36 suspension was not further diluted prior to injection.

### Screening the collection of miRNA allelic mutants

Two to three-day-old female flies were anesthetized with CO_2_, and 13.8 nl of *C. albicans* inoculant (∼800 yeast cells) injected into the dorsolateral region of the thorax using a Nanoject II Injector (Drummond Scientific) coupled to a fine glass needle. Flies were kept at 30° following infection; a compromise between conditions for *C. albicans* growth and fly resistance to temperature. Survival was monitored every 24 hr over a 3-day period; lethality during the first 6 hr was attributed to effects associated with the injection procedure and was subtracted from the survival data. The miRNA allelic mutants were infected as several groups with each group including a background control, either *w* or *yw*. The survival profile of a particular mutant background was compared to that of the group control via the Log-rank test, and *P*-values adjusted for multiple comparisons using the Holm–Šidák method. For miRNA mutants whose survival profile statistically separated from their group control, a second round of *C. albicans* infection was performed; for further comparison, additional backgrounds were included that did not separate from their group control. In addition, most second-round miRNA mutant backgrounds were injected with 13.8 nl PBS and selected with 13.8 nl *E. coli*. All confirmed second-round miRNA mutants were tested for a third time, confirming the differences in estimated survival probabilities found previously.

To estimate changes in pathogen number during the course of infection six, infected female flies were homogenized in SGB at specific time points (0, 24, and 48 hr); the homogenates were serially diluted, plated on SGA, and incubated for 36 hr at 30° to enable suitable colony growth for counting.

To determine changes in the levels of *Drs* and *Immune-induced Molecule 1* (*IM1*) transcripts during the course of infection, total RNA was extracted from six female flies at specific time points (0, 24, and 48 hr) using the Total RNA Purification Plus Kit (Cat. No. 48400; Norgen Biotek); this uses a column that efficiently removes genomic DNA. Purity and concentration of the RNA samples were checked using a NanoDrop 1000 spectrophotometer (Thermo Fisher Scientific), and 500 ng of RNA reverse transcribed in a total volume of 20 µl using the SensiFAST cDNA Synthesis Kit (Cat. No. BIO-65054; Bioline, London, UK). Template cDNA—2 µl diluted 10-fold—was amplified using the SensiFast SYBR No-ROX Kit (Cat. No. BIO-98005; Bioline) and primer pairs against *Drs*, *IM1*, or *TATA-Binding Protein* (*dTBP*) at a concentration of 400 nM per primer; cycling was performed in a Rotor-Gene Q real-time PCR machine (QIAGEN, Valencia, CA). The amount of transcript was expressed as 2^(−ΔCT)^, where ΔCT is the average threshold cycle (CT) value for the gene of interest (*Drs*/*IM1*) subtracted from the average CT value for the internal control (*dTBP*) (Schmittgen and Livak 2008). Primer pairs (5′–3′): *Drs* (+) GTACTTGTTCGCCCTCTTCG and *Drs* (−) TTAGCATCCTTCGCACCAG; *IM1* (+) GAAATTCTTCTCAGTCGTC and *IM1* (−) CATCAATGGCGATTGCAG; and *dTBP* (+) GGCAAAGAGTGAGGACGACT and *dTBP* (−) GAGCCGACCATGTTTTGAAT.

### Identifying potential NF-*κ*B-binding sites in close proximity to miRNA genes

The current version of the *D. melanogaster* genome (R6.12) was used to perform the binding site analysis. The transcription start sites (TSSs) for miRNA genes are yet to be comprehensively described, but predicted or experimentally determined TSSs generally lie within 1.5 kb of the *miR-RM* transcript (Qian *et al.* 2011; Ryazansky *et al.* 2011); these are the pre-miRNAs embedded in the primary transcripts. Therefore, the initial base (+ 1) of the *miR-RM* transcript was used as a reference for the promoter region of the corresponding miRNA gene. A Position Weight Matrix (PWM) approach was taken to identify potential NF-κB-binding sites within 2-kb regions flanking the (+ 1) of the *miR-RM* transcript; evidence from a study using a human cell line identified functional NF-κB-binding sites upstream and downstream of certain miRNA genes, and these sites were located up to 3 kb from the TSS (Zhou *et al.* 2010). The Toll and IMD PWMs were constructed using 15 sequences identified as overrepresented motifs upstream of Toll and IMD responsive genes, respectively (Busse *et al.* 2007). The Rel PWM was constructed from 31 sequences derived from a SELEX assay using a recombinant fragment of the *D. melanogaster* Rel protein, and the DIF/Rel PWM constructed from the base frequency table derived from a SELEX assay using a combination of recombinant DIF and Rel proteins (Senger *et al.* 2004).

### miRNA profiling

To identify changes in the miRNA profile over time, a microarray experiment was performed. Wild-type flies from the same population were injected with 13.8 nl of either *C. albicans*, *S. aureus*, *E. coli*, or PBS, and transferred to 30°. At 0, 1, 7.5, 18, and 60 hr following injection, total RNA was purified from 10 females using the Total RNA Purification Plus Kit (Cat. No. 48400; Norgen Biotek). Next, 5 μg of total RNA (quantified using a NanoDrop 1000 spectrophotometer) from each sample was suspended in 200 μl of precipitation solution (3 M NaOAc, pH 5.2 and 100% ethanol) and immediately frozen in liquid nitrogen; these were stored at −80° until shipment for the miRNA microarray experiment (LC Sciences, Houston, TX). Samples were labeled with a fluorescent dye and hybridized to a µParaflo Microfluidic Chip that contained complementary sequences for 425 unique mature miRNA sequences, derived from 241 *D. melanogaster* miRNA genes (miRBase version 19.0). The background signal was subtracted and data normalized prior to analysis; ANOVA was applied to identify statistical differences. All data processing was performed by LC Sciences and is presented as clustered heat maps. Experiments were repeated independently on three occasions (*n* = 3), except for the *E. coli* infection, which was performed twice (*n* = 2).

### Quantification of branched-chain amino acids (BCAAs)

Ten flies injected with PBS or *C. albicans* (weighing 10 mg) were flash-frozen in liquid nitrogen at time 0–24 hr following injection. Frozen flies were homogenized in 200 µl assay buffer. Homogenates were centrifuged for 10 min at 12,000 rpm, and 10 µl of supernatant used to measure the BCAA levels (BCAA Colorimetric Assay Kit; Cat. No. K564-100; Biovision); color intensity was quantified using a microplate reader (CLARIOstar; BMG Labtech).

### Statistical analysis

Data were analyzed using GraphPad Prism (version 7.01) or the R-platform. To visualize survival data, estimated survival curves were constructed; in figures with estimated survival probabilities, C.I.s were excluded for clarity of image. The Log-rank test was used to identify statistical differences between survival curves and *P*-values were adjusted for multiple comparisons via the Holm–Šidák method. Data involving a time series (excluding the microarray data) were analyzed using repeated-measures two-way ANOVA, and *P*-values adjusted for multiple comparisons using Bonferroni correction. Single time point data were compared via one-way or two-way ANOVA, and *P*-values adjusted using the Holm–Šidák method.

### Data availability

The miRNA mutant strain collection from the Cohen Lab and the mir sponge lines are available from the Bloomington *Drosophila* Stock Center. All *P*-values for Figure 6 can be found in Supplemental Material, Table S2. The microarray data are presented in Figure 4 for miRNA that presented statistically significant regulation and in Figure S3 for all others; Table S5 includes a list of *P*-values for the microarray analysis. Table S4 has a list of all miRNAs that have at least one consensus NF-κB-binding site, while Figure S4 summarizes the numbers of miRNAs with such site(s).

## Results

### Disrupting miRNA biogenesis causes susceptibility to systemic C. albicans infection and potentially influences Toll signaling

To explore the contribution of miRNAs in controlling the response to infection at a whole-organism level, we initially examined the requirement for miRNA biogenesis in protecting the host against infection. To perturb this process, we used RNAi against Exp5, which transports pre-miRNAs from the nucleus to the cytoplasm, and against Loqs and Dcr-1, which together process the various pre-miRNAs to produce particular miRNA duplexes that are subsequently loaded onto Ago-1. We also examined the consequences of increasing the levels of Ago-1, since this protein is involved in production of the miRNA strand that guides translational repression/destabilization of target mRNAs (Ha and Kim 2014). Manipulating these components has been shown to alter the miRNA profile in human cell lines (Lee *et al.* 2004; Okamura *et al.* 2004; Hata and Kashima 2016; Jakob *et al.* 2016; Kim *et al.* 2016).

**Figure 1:**
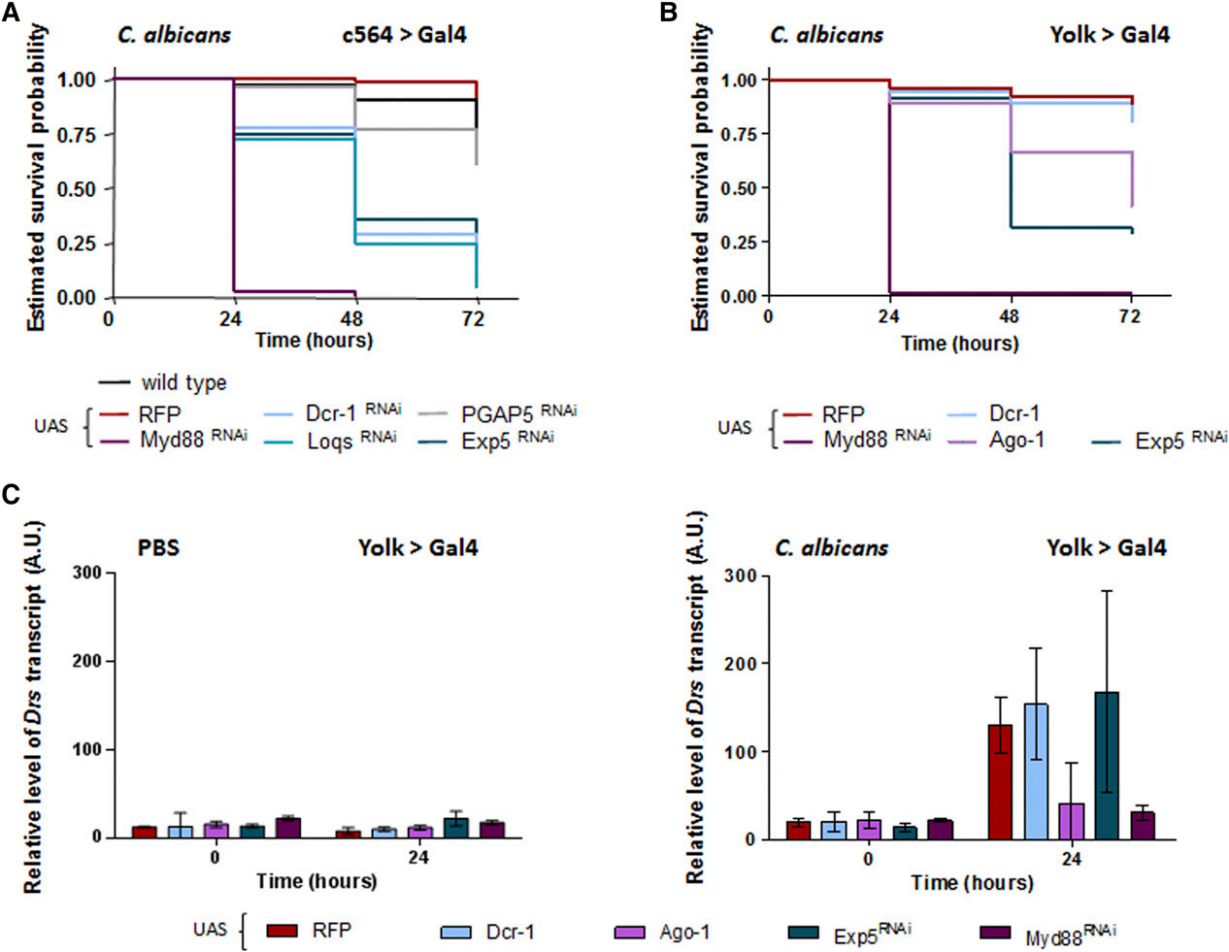
Perturbing miRNA biogenesis increases the susceptibility of flies to systemic *C. albicans* infection and affects production of the *Drs* transcript. (A) RNAi knockdown of Exp5, Dcr-1, or Loqs in the major immune tissues results in flies becoming more susceptible to systemic *C. albicans* infection relative to a wild-type strain, or RFP and PGAP5 controls, but they are less susceptible than immune-defective flies; *P* < 0.001 for the wild-type strain or either control compared to any miRNA biogenesis deficient background, and when the latter are compared to the Myd88 mutant. The survival profile of the wild-type strain is inseparable to either control (*P* > 0.1 in both cases). (B) Likewise, when Exp5 function is reduced or Ago-1 overexpressed specifically in the fat body, flies become more sensitive to systemic *C. albicans* infection relative to an RFP control (*P* < 0.001 in both cases), but are less sensitive than Myd88-deficient flies (*P* < 0.001 in both cases). The survival profiles for Yolk > Dcr-1 and control RFP flies are inseparable (*P* > 0.2). For clarity of image, 95% C.I.s have been omitted. (C) At the point of injection (time 0), there are generally no differences in the level of *Drs* transcript (PBS injected, *P* > 0.6 for any comparison, except for those with Yolk > Myd88^RNAi^ flies where the transcript is slightly elevated; *C. albicans* injected, *P* > 0.05 for any comparison), implying that disrupting miRNA biogenesis *per se* does not alone affect its production. The amount of transcript is inseparable for Yolk > Ago-1 and Yolk > Myd88^RNAi^ flies 24 hr following infection (*P* > 0.9), and does not detectably change for either from the point of injection (*P* > 0.9 in both cases). However, the level of *Drs* transcript increases during the course of infection within Yolk > Dcr-1 and Yolk > Exp5^RNAi^ flies (*P* < 0.001 in both cases) to that of the RFP control (*P* > 0.2 for both comparisons to control). Only Yolk > Exp5^RNAi^ flies exhibit a change in *Drs* transcript over time from the point of PBS injection (*P* < 0.01). Survival profiles were compared using the Log-rank test (*n* = 35–104, combined data from three independent infections) and *P*-values adjusted for multiple comparisons via the Holm–Šidák method. Quantification of *Drs* transcript was performed independently three times (*n* = 3) and is presented as mean values with 95% C.I.s; differences in relative transcript level were identified using repeated-measure two-way ANOVA and *P*-values adjusted for multiple comparisons through Bonferroni correction. miRNA, microRNA; RNAi, RNA interference; RFP, red fluorescent protein; UAS, upstream activating sequence; 95% CI, 95% Confidence interval.

The c564 > Gal4 driver was used to express UAS-RNAi constructs in the major immune-responsive tissues (fat body, hemocytes, and gut) throughout development and into adulthood (Hrdlicka *et al.* 2002; Ryu *et al.* 2006). Female c564 > RNAi flies were injected with either *C. albicans* or PBS—the latter serving as a control for the injection procedure—and survival monitored. None of the c564 > RNAi flies exhibited physical abnormalities, nor did they die significantly following injection with PBS (data not shown). Those impaired for miRNA biogenesis (c564 > Exp5^RNAi^/c564 > Loqs^RNAi^/c564 > Dcr-1^RNAi^) succumbed to systemic *C. albicans* infection more rapidly than a wild-type strain (see *Materials and Methods*), and more rapidly than flies expressing either red fluorescent protein (RFP) (c564 > RFP) or a control RNAi construct (c564 > PGAP5^RNAi^) (Figure 1A). PGAP5 was chosen as a control since it has no defined role in immunity—likewise regarding the vertebrate ortholog metallophosphoesterase-1—and did not exhibit resistance/tolerance or susceptibility to *C. albicans* infection during a large-scale UAS-RNAi screen (data not shown); consistent with this, the survival of c564 > PGAP5^RNAi^ flies was inseparable from the wild-type strain or from those expressing RFP (Figure 1A). However, flies impaired for miRNA biogenesis were less susceptible to infection than those deficient in Toll signaling (c564 > Myd88^RNAi^) (Figure 1A). These observations suggest that having the correct complement of miRNAs within immune tissues is necessary for flies to cope with systemic yeast infection. It may therefore be that dysfunctional miRNA biogenesis affects the output of Toll signaling, the major respondent to fungal infections. To examine this, we used Yolk > Gal4 to overexpress Ago-1 or Dcr-1 protein, or the Exp5^RNAi^ construct, specifically in the fat body of female flies (Yolk > Ago-1/Yolk > Dcr-1/Yolk > Exp5^RNAi^); the driver is expressed ∼4 days after emergence of the adult and therefore bypasses potential effects that may occur during development (Georgel *et al.* 2001). Flies were again injected with either PBS or *C. albicans* and a subset of each population assessed for survival; the level of *Drs* transcript was measured in the surviving populations to determine an output of Toll signaling. There were no significant differences in the survival dynamics for flies injected with PBS (data not shown). Consistent with that observed with c564 > Exp5^RNAi^, Yolk > Exp5^RNAi^ flies succumbed to systemic *C. albicans* infection more rapidly than those of the control population (Yolk > RFP), but not as rapidly as those deficient for Toll signaling (Yolk > Myd88^RNAi^) (Figure 1B). The survival profile for Yolk > Ago-1 flies was similarly positioned, and inseparable to that of Yolk > Exp5^RNAi^ flies (Figure 1B). Although susceptibility to infection was equivalent, there were differences in their level of *Drs* transcript: for Yolk > Ago-1 flies this did not detectably change over the course of infection—mirroring that observed with a Toll pathway mutant—but it increased within Yolk > Exp5^RNAi^ flies comparably to that observed with the Yolk > RFP control (Figure 1C). An additional feature unique to Yolk > Exp5^RNAi^ flies was the significant increase relative to the control of the *Drs* transcript induced through PBS injection alone (Figure 1C).

Therefore, it seems likely that correct processing of miRNAs within immune tissues is necessary to protect against systemic *C. albicans* infection and, in some capacity, to control the output of Toll signaling. To identify specific miRNAs that contribute to either function, we used a collection of targeted knockout miRNA mutants recently constructed in *D. melanogaster* (Chen *et al.* 2014).

### Screening for miRNAs that contribute to controlling the fly’s response to systemic C. albicans infection

**Figure 2:**
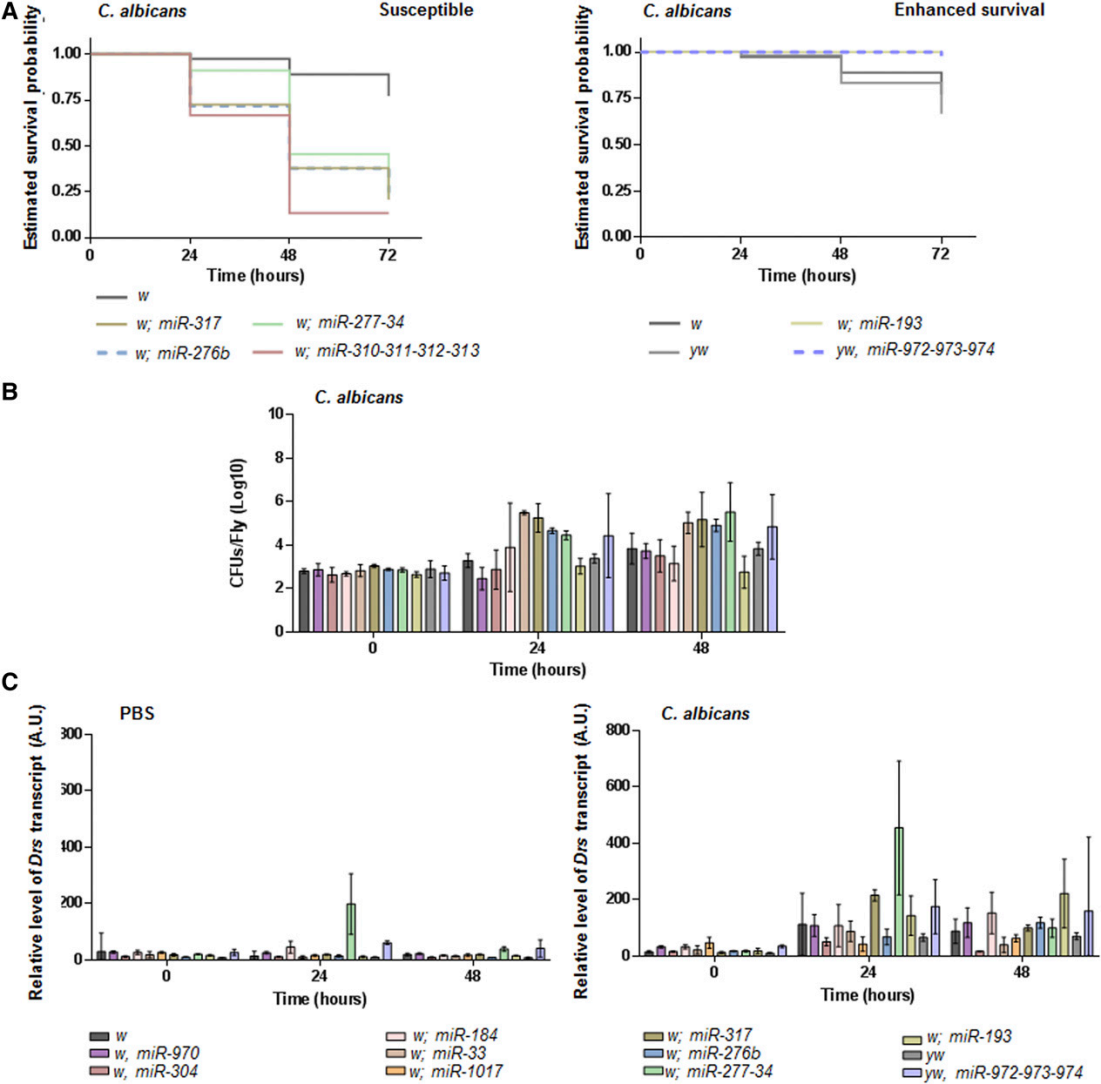
Assaying selected miRNA allelic mutants for characteristics indicative of defects to immune signaling. (A) Survival profiles for miRNA mutant backgrounds shown to exhibit susceptibility or enhanced survival in response to systemic *C. albicans* infection; the estimated survival curves represent combined data for the repeat infections (*n* = 29–62, see Table S1). The *w* and *yw* curves were constructed using survival data taken from the groups within which a miRNA mutant background was originally compared: for *w* this is combined data from five groups (*n* = 153), and for *yw* combined data from two groups (*n* = 54). Survival profiles for the miRNA mutants separate significantly from their given *w* or *yw* control (*P* < 0.001 for any mutant compared to control). For clarity of image, 95% C.I.s have been omitted. (B) Changes in pathogen number during the course of systemic *C. albicans* infection. The initial inoculation (time 0) is equivalent for all miRNA mutants and the *w* and *yw* controls (*P* > 0.05 for any comparison). (C) Changes in the level of *Drs* transcript following PBS injection or infection. For each miRNA mutant, conclusions regarding the dynamics of the two variables over time and how this compares to the given control are summarized in Figure 6. CFUs and *Drs* quantification were performed independently on three occasions (*n* = 3) and are presented as mean values with 95% C.I.s. CFU, colony forming unit; miRNA, microRNA; 95% CI, 95% Confidence interval.

To date, the miRNA mutants generated by the Cohen Lab have been assayed for changes in a variety of phenotypes but not their response to infection (Chen *et al.* 2014). Therefore, we injected the majority of available lines with *C. albicans* and compared their survival profiles to that of a background control: in total, we evaluated 72 miRNA mutant backgrounds covering 95 miRNAs. For miRNA mutants whose survival profile statistically separated from their control (*P* < 0.05), two repeat rounds of injection were performed; included in this were mutants whose survival profiles were close to but did not separate from their control (0.1 ≥ *P* > 0.05), and for further comparison, additional lines whose survival profiles were inseparable from their control (*P* > 0.1) (Table S1). We identified four mutant backgrounds that were more susceptible (*miR-317*, *miR-276b*, *miR-277-34*, and *miR-310-311-312-313*) to systemic *C. albicans* infection, and two mutant backgrounds that exhibited enhanced survival (*miR-193* and *miR-972-973-974*) (Figure 2A and Table S1). In addition, none of the flies carrying the *miRNA-137* mutation succumbed to infection (see Table S1); however, due to the low number of obtainable flies at the time of injection, the enhanced survival was not considered significant. Finally, none of the flies for the six mutant backgrounds exhibiting altered survival profiles had obvious defects to their external morphology, as all appeared healthy, nor did they exhibit differences in survival dynamics following PBS injection (Table S1).

We next assessed the ability of the six miRNA mutants to control pathogen number by quantifying their colony forming units (CFUs) over time following inoculation with *C. albicans*, and for their ability to modulate an output of Toll signaling. These are two assays that reveal defects in classic innate immune function, and regarding CFUs, distinguish between mechanisms of resistance and tolerance toward infection (Schneider and Ayres 2008). For comparison, we also evaluated some of the miRNA mutants whose survival profiles were inseparable from their controls. During the first 24 hr, pathogen number increased within the susceptible miRNA mutants to a level greater than that observed with the *w* background control (Figure 2B and Figure 6). For the *miR-317* and *miR-276b* susceptible mutants, pathogen number did not detectably change during a further 24 hr of infection, but continued to increase within the *miR-277-34* mutant; at this 48-hr time point there was more *C. albicans* within the susceptible miRNA mutants than the *w* control (Figure 2B and Figure 6). For miRNA backgrounds whose survival did not differ from the *w* control there was, in general, no separation of pathogen number (Figure 2B and Figure 6). The most notable exception to this being for flies carrying the *miR-33* mutation, whose CFU profile was akin to that of the susceptible mutants (Figure 2B and Figure 6). Within the *miR-972-973-974* mutant, which exhibited enhanced survival, the amount of *C. albicans* significantly increased over time relative to the *yw* background control, for which there was no detectable change in CFUs (Figure 2B and Figure 6). Finally, miR-193 mutants that exhibited enhanced survival did not show any significant change from the initial inoculate over the 48-hr period (Figure 2B and Figure 6).

There were significant differences in the levels of *Drs* transcripts between some of the miRNA mutants at the point of injection (time 0 PBS and time 0 *C. albicans*, Figure 2C). However, for mutants carrying a *w* allele, these were inconsistent between those injected with PBS and those injected with *C. albicans*—the assumption being that differences should be consistent at time 0 if there is an effect due to mutation—and thus likely reflect natural variation rather than meaningful differences resulting from inherent biological changes. An exception to this may be the *miR-304* mutant, where the level of transcript was reproducibly low across the course of infection (Figure 2C and Figure 6); this suggests that production of *Drs* mRNA may actually be compromised. All miRNA mutants (except *miR-1017*) and both controls experienced an elevation in their levels of *Drs* transcripts 24 hr following systemic *C. albicans* infection. For the susceptible *miR-317* and *miR-277-34* mutants and enhanced survival *miR-972-973-974* mutant, this was above that of the respective *w* and *yw* control, being particularly notable for *miR-277-34*; these increases resolved to the level of control during the subsequent 24-hr infection period (Figure 2C and Figure 6). At this 48-hr time point, miRNA mutants with survival profiles similar to their control had either maintained their elevated levels of *Drs* transcripts (*miR-970* and *miR-184*) or experienced a decrease to levels below that of the *w* background (*miR-304* and *miR-33*) (Figure 2C and Figure 6). The *miR-276b* and *miR-193* mutants continued to increase the level of transcript over the 48-hr infection period; for the susceptible *miR-276b* mutant this was at a level comparable to the *w* control, whereas the enhanced survival *miR-193* mutant was accumulating significantly more (Figure 2C and Figure 6). For flies injected with PBS, only the susceptible *miR-277-34* and enhanced survival *miR-972-973-974* mutants exhibited a significant increase in the levels of *Drs* transcripts 24 hr following injection, and this was above that of the respective control (Figure 2C and Figure 6). The *miR-972-973-974* mutant also had elevated transcript levels at the point of injection, suggesting that basal production of *Drs* is, to some degree, heightened in this mutant.

It is clear that individual or small clusters of miRNAs contribute to control of the response to systemic *C. albicans* infection through modulating the survival outcome, in general, limiting the increase in pathogen number, and influencing the production of the *Drs* transcript. These are traits associated with changes to Toll signaling. Therefore, we explored this potential connection.

### miR-277 and miR-34 potentially have opposing effects on the output of Toll signaling

**Figure 3:**
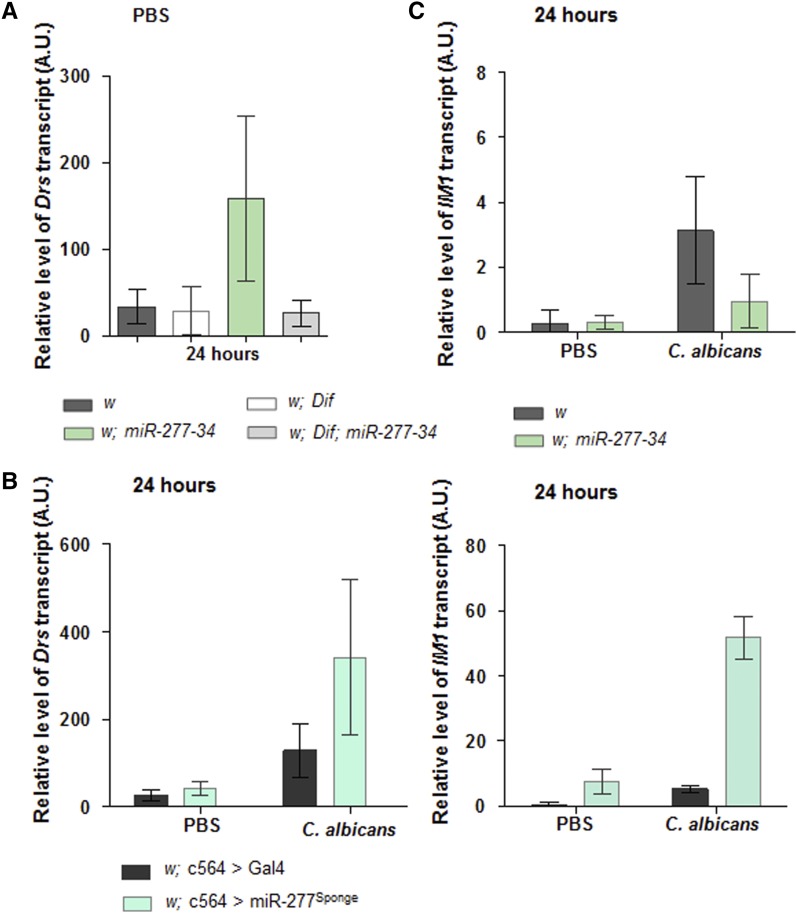
miR-277 and miR-34 potentially influence signaling through the Toll pathway. (A) When Dif function is also compromised, the elevation of *Drs* transcript observed within the *miR-277-34* mutant 24 hr following PBS injection is abolished; *P* < 0.001 for any comparison to *w*; *miR-277-34*, and *P* > 0.9 for all other comparisons. (B) Knocking down the function of miR-277 in immune tissues (c564 > miR-277^Sponge^) leads to an increase in the level of *Drs* transcript 24 hr following infection relative to the control (c564 > Gal4, this is the driver crossed to the *w* background, *P* < 0.001), and for *IM1* transcript, this is true also for PBS injection (*P* < 0.001 for PBS injection and infection). (C) In contrast to that observed with *Drs*, the level of *IM1* transcript within the *miR-277-34* mutant is lower than that of the control 24 hr following infection (*P* < 0.001). The data represents the mean of three independent biological repeats (*n* = 3/95% C.I.s) and was compared using one-way ANOVA (A) or two-way ANOVA (B and C); *P*-values were adjusted via the Holm–Šidák method. 95% CI, 95% Confidence interval.

A defining feature of the susceptible *miR-277-34* mutant is the strong *Drs* response observed 24 hr following PBS injection. We eliminated the increase in temperature—applied since PBS-injected flies serve as the control for infection, which proceeds at 30°—as being a causal factor, and demonstrated that sterile pricking is sufficient to invoke the response (Figure S1). This implies participation of Toll/IMD signaling, since pricking alone induces rapid production of numerous AMP transcripts at a low level (Lemaitre *et al.* 1997); presumably, in the *miR-277-34* mutant, this pricking effect is not so efficiently downregulated. To substantiate a requirement for Toll signaling, we repeated the PBS injection of the *miR-277-34* mutant while simultaneously reducing the function of Dif, the major Toll pathway transcription factor in the context of immunity. This suppressed the strong *Drs* response (Figure 3A), indicating that Toll signaling is required to produce the pool of transcript modulated by miR-277 and/or miR-34. To evaluate whether miR-277 alone was sufficient to regulate the level of *Drs* transcript in response to PBS injection and/or infection, we used an antisense RNA to knock its function down within the immune tissues (c564 > miR-277^Sponge^). For an independent readout of Toll signaling, we also quantified the relative level of *IM1*, a gene whose infection response is exclusively dependent on the Toll ligand Spz (De Gregorio *et al.* 2001, 2002). Although 24 hr following PBS injection the level of *Drs* transcript was slightly elevated within c564 > miR-277^Sponge^ flies compared to those of the control, the difference was not significant; however, there was significantly more transcript following infection (Figure 3B). The level of *IM1* transcript was higher than that observed for the control population 24 hr following either PBS or *C. albicans* injection (Figure 3B); an effect equivalent to that observed with *Drs* transcript in the *miR-277-34* mutant (Figure 2C). This suggests that miR-277 functions to reduce signaling through the Toll pathway, and that its loss in the *miR-277-34* mutant may be the causal factor underlying the response to infection. However, if a repression of Toll signaling has been removed, it raises a question regarding the susceptibility of the mutant. Therefore, we measured the level of *IM1* transcript 24 hr following PBS injection or infection; in contrast to *Drs*, the level of transcript was comparable to the control following injection of PBS, and significantly lower in response to infection (Figure 3C). This suggests that miR-34 functions to promote signaling through the Toll pathway and acts downstream to the inhibitory effect of miR-277. The elevated level of *Drs* transcript within the *miR-277-34* mutant, if signaling through Toll is reduced, may be a consequence of either miR-277 and/or miR34 directly targeting the transcript. Indeed, members of the *miR-310-311-312-313* cluster and *miR-964* target the 3′-UTR of *Drs* to reduce transcript abundance (Vodala *et al.* 2012; Li *et al.* 2017b). Therefore, we used freely available algorithms—PITA/TargetScan/miRanda-mirSVR (Enright *et al.* 2003; Kertesz *et al.* 2007; Ruby *et al.* 2007; Betel *et al.* 2010; Agarwal *et al.* 2015)—to scan the 3′-UTR of *Drs* and identified a potential binding site for miR-277 (Table S3), suggesting direct targeting of the transcript as a potential regulatory mechanism. There were no potential sites for miR-277 in the 3′-UTR or open reading frame (ORF) of *IM1*. The latter may be significant given that conserved miRNA-binding sites in the ORF of *D. melanogaster* mRNAs lead to downregulation of transcript levels (Schnall-Levin *et al.* 2010). There were also no potential binding sites for miR-34 in either the *Drs* or *IM1* transcripts (Table S3). Therefore, we speculate that loss of miR-277 in the *miR-277-34* mutant results in derepression of accumulated *Drs* transcript, and that this compensates for reduced signaling through the Toll pathway due to loss of miR-34.

From the miRNA mutants assessed for traits indicative of defects in innate immunity (Figure 6), other than miR-277, only miR-33 and miR-972 have potential binding sites in the 3′-UTR of the *Drs* transcript; none of the miRNAs are predicted to target its ORF (Table S3). This is consistent with studies demonstrating that miRNAs function in positive or negative feedback loops that modulate signaling through NF-κB pathways (Olarerin-George *et al.* 2013; Staedel and Darfeuille 2013; Teng *et al.* 2013; Maudet *et al.* 2014; Kumar *et al.* 2015; Xiong *et al.* 2016; Li *et al.* 2017b). Accordingly, immune-induced expression (or repression) of particular miRNAs has been shown to depend on NF-κB-binding sites (Taganov *et al.* 2006; O’Hara *et al.* 2010; Zhou *et al.* 2010; Wang and Cambi 2012; Thompson *et al.* 2013). In an attempt to identify miRNAs regulated by NF-κB activity, we assessed the potential for NF-κB occupancy in proximity to miRNA genes, and evaluated changes in the miRNA profile following systemic infection.

### Distribution of potential NF-*κ*B sites in proximity to miRNA genes and miRNA microarray analysis following systemic infection

A PWM approach was used to identify potential NF-κB-binding sites within 2-kb regions located either side of the first base (+) of the *miR-RM* transcripts; four PWMs were constructed (Table S4). Of the 256 miRNA genes in the annotated *D. melanogaster* genome, 36% had at least three strong NF-κB-binding sites (Figure S2), suggesting that certain miRNAs may indeed be regulated by innate immune signaling at the level of transcription.

**Figure 4:**
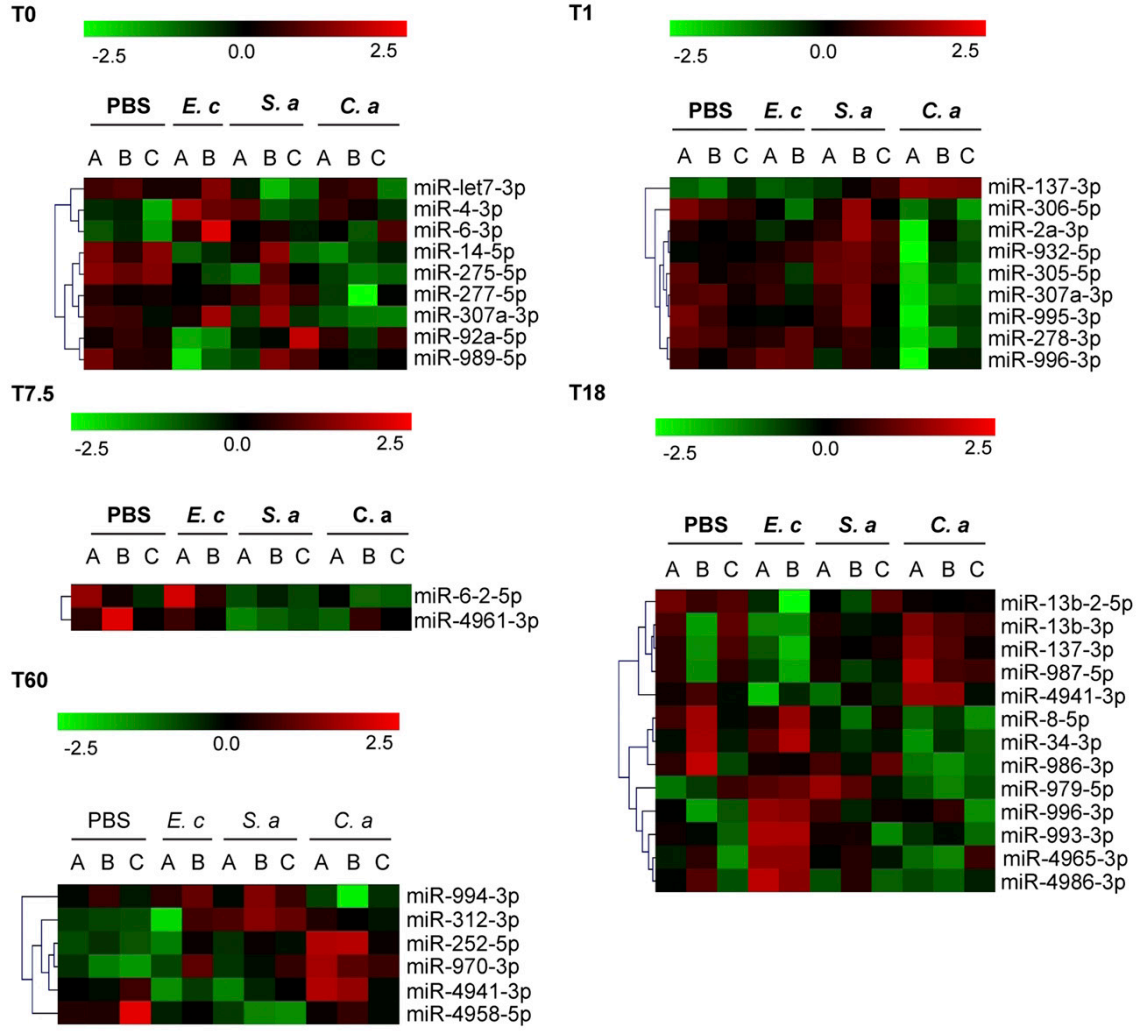
Differential production of mature miRNA forms. Wild-type flies were injected with PBS or one of the given pathogens: *E. coli* (*E. c*), *S. aureus* (*S. a*), or *C. albicans* (*C. a*). Their miRNA complement was quantified at time points T0, T1, T7.5, T18, and T60 (numbers refer to hours following injection). Data are compared for all factors at each individual time point to identify mature miRNA forms whose production is differentially regulated; ANOVA-selected miRNAs (*P* < 0.1) are presented as clustered heat maps, where negative *Z*-values (green) indicate downregulation relative to the data set and positive *Z*-values (red) upregulation. Except with *E. coli* (*n* = 2), the infection experiments were repeated independently on three occasions (*n* = 3), indicated above the heat maps as A, B, and C. miRNA, microRNA.

To determine whether stimulating the Toll or IMD pathways altered the miRNA profile over time following systemic immune challenge, we performed a miRNA microarray experiment. Wild-type flies were infected with *C. albicans* or *S. aureus* (both Toll signaling), or *E. coli* (IMD signaling); the baseline was PBS injection. Samples were collected at time points (0, 1, 7.5, 18, and 60 hr following injection) a few hours prior to when significant changes occurred in Toll/IMD signaling, the assumption being that modulation of the miRNA profile must precede regulation of mRNAs. There were changes (*P* < 0.1, an acceptable threshold for exploratory data) in the profile over time for 38 and 33 mature miRNAs following *C. albicans* or *S. aureus* infection, respectively, and 15 for PBS injection (Figure S3 and Table S5). Of note, 13 of those modulated by *S. aureus* infection were also affected by systemic *C. albicans* (Table S5), supporting the notion their expression may indeed be regulated by Toll signaling. However, when PBS and pathogen were compared at the particular time points (Figure 4), from the 13 mature forms, only miR-275-5p and miR-307a-3p (time 0), and miR-305-5p/miR-306-5p/miR-307a-3p (1 hr) significantly separated (Table S5), and this was mostly due to there being lower levels in response to *C. albicans* infection. Among the miRNA profiles that changed over time were some required to control the response to systemic *C. albicans* infection (Figure 6, and here we include *miR-137*): *C. albicans* (miR-34-5p/miR-137-3p/miR-313-5p); *S. aureus* (miR-34-5p/miR-277-5p/miR-310-3p and miR-310-5p/miR-311-3p); and PBS (miR-277-5p). However, again, these differences did not come through strongly at the particular time points; in fact, only mature form miR-137-3p was shown to separate, being higher at two time points following *C. albicans* infection. In addition, miR-312-3p was at a higher level (60 hr) and miR-34-3p at a lower level (18 hr) in response to *C. albicans* and *S. aureus* infection. The signal intensity values for miR-34 have been plotted as an example of what the data generally looks like (Figure S4). This highlights three observations: (1) miRNAs whose profiles change significantly across time for a particular factor tend to do so at the later time points and (2) do not separate so much from the PBS baseline (miR-34-5p); and (3) where a difference is found between PBS and pathogen at a particular time point it tends to result from small error between biological repeats (miR-34-3p).

### Systemic C. albicans infection promotes degradation of BCAAs

To assess potential functions of the miRNAs shown to contribute in controlling the response to systemic infection, we used DIANA TOOLS mirPath v.3 to predict the pathways and genes they are likely to regulate (Vlachos *et al.* 2015) (Table S6). Three of the most striking predictions were for miR-972 in modulating Hippo signaling and endocytosis, and for miR-277 in regulating catabolism of BCAAs (valine, leucine, and isoleucine); these are important nutrient signals that affect metabolism, for example, through activating the mammalian target of rapamycin complex 1 or serving as substrates for the tricarboxylic acid (TCA) cycle (Yoon 2016). Given that miR-277 does indeed regulate BCAA catabolism in *D. melanogaster* (Esslinger *et al.* 2013), we first examined whether infection can affect the process, and second whether this is modified in the *miR-277-34* mutant.

**Figure 5:**
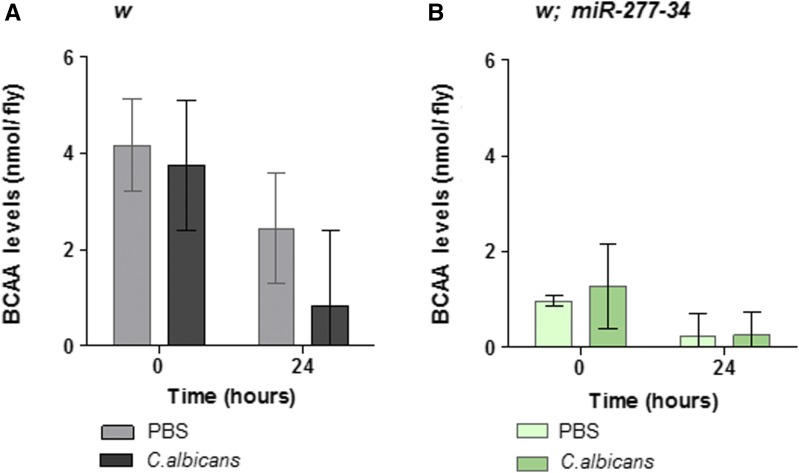
Systemic *C. albicans* infection contributes to lowering the total level of BCAAs. The total level of BCAAs within the *w* background and *miR-277-34* mutant were measured at 0–24 hr following injection of PBS or *C. albicans*. (A) There is a decrease over time when *w* flies are injected with either PBS or *C. albicans* (*P* < 0.01 in both cases), and this resolves to a significant difference in levels at 24 hr, being more extensive following infection (*P* < 0.01). (B) This is similar to that observed with the *w* background (over time *P* < 0.05 in both cases), except there is no difference in BCAA levels come 24 hr (*P* > 0.2). Data presented as the mean of three independent biological experiments (*n* = 3/95% C.I.s); repeated-measure two-way ANOVA and Bonferroni correction were applied to determine statistical differences. BCAA, branch-chained amino acid; 95% CI, 95% Confidence interval.

We injected *w* control flies and those of the *miR-277-34* mutant with PBS or *C. albicans* and quantified their BCAA content after 24 hr. This decreased significantly in control flies during the 24-hr infection period whether they were injected with PBS or infected (Figure 5); we attribute the former to changes in metabolism that are likely to occur as a result of keeping flies at 30° during the infection period or alternatively, as a result of the injection *per se*. Nevertheless, within control flies the BCAA content was significantly lower following infection relative to PBS injection after 24 hr (Figure 5). In contrast, within the *miR-277-34* mutant, BCAA levels were indistinguishable at 24 hr (Figure 5), although reduced compared to the levels at the point of injection (time 0, Figure 5). However, we note that *miR-277-34* mutant flies had a significantly lower BCAA content relative to the *w* control at the point of time of injection (time 0, Figure 5). This suggests that BCAA catabolism was elevated in the mutant in the absence of infection, consistent with a previous study that demonstrated that overexpression of *miR*-277 negatively impacted on BCAA catabolism (Esslinger *et al.* 2013). Therefore, we propose that BCAA catabolism is constitutively active in the *miR-277-34* mutant, and that resolvable differences relating to infection are consequently lost. However, the data demonstrate that systemic infection stimulates the catabolism of BCAAs.

## Discussion

In animal tissues, miRNAs regulate many aspects of innate and adaptive immunity, functioning within a variety of network motifs that provide robustness to biological processes through buffering or setting thresholds for protein production or cell signaling (Ebert and Sharp 2012; Chen *et al.* 2013; Mehta and Baltimore 2016). Here, we have used *D. melanogaster* as a model system to explore the relationship between miRNAs and the response to systemic infection at the whole-organism level.

### The miRNA biogenesis pathway

We first observed that RNAi knockdown of Exp5 or overexpression of Ago-1, manipulations that alter the miRNA profile of human cell lines (Maudet *et al.* 2014; Hata and Kashima 2016), led to immune deficiency phenotypes. Although survival profiles were indistinguishable when either component was manipulated specifically in fat body tissue, differences were seen regarding their effect on the level of *Drs* transcript; with Ago-1 overexpression, *Drs* was not induced in response to infection—behaving equivalently to a Toll pathway mutant—whereas *Drs* transcript level was comparable to the control with RNAi knockdown of Exp5. This suggests that the mechanism through which they exert their effect is different. One possibility is that Ago-1 impacts directly on a component of the Toll pathway, as has been described for Dcr-2, a key factor in the biogenesis of small interfering RNAs (Wang *et al.* 2015). These authors showed that Dcr-2 binds directly to the mRNA of *Toll* and promotes production of the receptor; consequently, Dcr-2 mutant flies are more sensitive to infection and exhibit reduced stimulation of Toll. The fact that knockdown of Exp5 causes enhanced susceptibility to systemic *C. albicans* infection, without appearing to affect the output of Toll signaling, suggests that other factors are dysregulated that otherwise contribute to promoting survival; this may be a consequence of broad changes to the miRNA profile leading to widespread dysfunction of host physiology.

### Immune phenotypes of miRNA mutants

**Figure 6:**
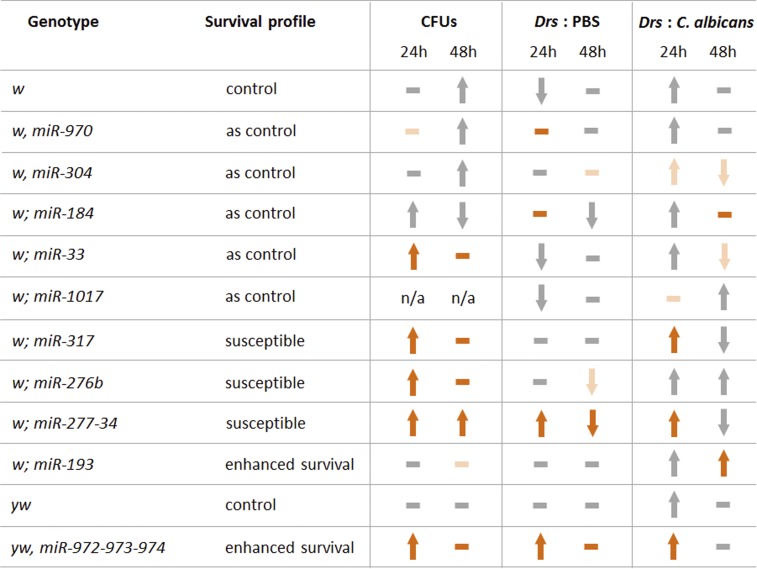
Summary of phenotypes for selected miRNA mutant backgrounds. The table presents a summary for each miRNA mutant regarding changes to pathogen number (CFUs) and level of *Drs* transcript over time (see Figure 2, B and C). Direction of arrows in the CFUs column and both *Drs* columns (PBS and *C. albicans*) indicate how the measured variable changes over time for the particular miRNA background; a dash indicates there was not a significant change. The arrow/dash color represents whether the change is significantly greater (orange) than that of the given *w* or *yw* control (gray) or significantly below (light orange) that of the given control; if the variable does not separate significantly from the given control the arrow/dash is given as gray. For details regarding *P*-values see Table S2. CFU, colony forming unit; miRNA, microRNA; n/a, not applicable.

Our assessment regarding the contribution of individual or small clusters of miRNAs in regulating events during the infection process revealed that miR-277 and miR-34 are likely to have opposing effects on Toll signaling (Figure 6). Compromising the function of miR-277 within the immune tissues led to significant overproduction of the Toll-dependent transcripts *Drs* and *IM1* in response to systemic *C. albicans* infection, and regarding the latter, this was also true for PBS injection. Given that infection-induced production of the *IM1* transcript is exclusively dependent on the Toll receptor ligand Spz (De Gregorio *et al.* 2001, 2002), and that the transcript does not contain binding sites for miR-277 in its 3′-UTR or ORF, it seems most likely that Toll signaling is overactive in the absence of miR-277; this implies that the miRNA functions to reduce signaling through the pathway. Thus, the strong *IM1* response following PBS injection when miR-277 function is compromised (or strong *Drs* response following sterile injury of the *miR-277-34* mutant) may result from an inability to downregulate a known stimulation in the production of AMP transcripts that occurs early, and at a low level, in response to pricking alone (Lemaitre *et al.* 1997). This suggests that miR-277 may set a threshold to prevent robust stimulation of Toll signaling in response to low-level stimuli. In this regard, it could participate in a mechanism similar to that described for miR-146, which is proposed to regulate signaling through Toll-like receptor (TLR)4 at subinflammatory levels (Schulte *et al.* 2013). In contrast, production of *IM1* transcript within the *miR-277-34* mutant was comparable to that of the control following PBS injection, and actually compromised in response to infection, suggesting that miR-34 functions downstream of miR-277 to promote signaling through the Toll pathway. This would explain why the *miR-277-34* mutant is susceptible to infection and does not efficiently control pathogen number compared to a control strain. An explanation as to why the level of the *Drs* transcript is overproduced in this mutant following PBS injection or infection may be direct targeting of the transcript, since it has a putative binding site for miR-277. Therefore, in the absence of miR-34, signaling through the Toll pathway would be compromised but the *Drs* transcript would accumulate due to concomitant loss of miR-277-dependent degradation. An alternative explanation is that other dysregulated pathways converge on the Toll pathway to induce *Drs* expression. For example, in *Drosophila* S2* cell culture, stimulating the Ecdysone receptor in the presence of diaminopimelic acid-type peptidoglycan induces production of *Drs* mRNA, and this requires the transcription factor Br-C (Rus *et al.* 2013). Relevant here is the recent observation that overexpression of miR-34 leads to reduced abundance of the Br-C protein (Xiong *et al.* 2016). Therefore, it may be that the level of Br-C is elevated in the *miR-277-34* mutant, and that this is somehow provoked to induce the *Drs* transcript in response to pricking and/or infection.

One reason why miR-277 and miR-34 would exert an opposing influence on Toll signaling may be that they have coevolved to regulate the response to infection, since miR-277 originates from miR-34 (Marco *et al.* 2013). This is also true for miR-317, the mutant of which responds in a manner equivalent to that of the *miR-277-34* mutant regarding infection. The elevated level of the *Drs* transcript observed within the *miR-317* mutant in response to systemic *C. albicans* is consistent with data showing that overexpression of miR-317 leads to reduced production of the transcript following *Micrococcus luteus* inoculation, and with miR-317 being strongly predicted to target Pellino, a negative regulator of the Toll pathway (Ji *et al.* 2014; Li *et al.* 2017b). That miRNAs do indeed regulate signaling through the Toll pathway has been demonstrated for miR-958 (also identified in our microarray analysis), which when overexpressed in flies leads to a reduction in the levels of Toll and Dif protein and delays the immune response (Li *et al.* 2017a). Only within the *miR-193* mutant did the *Drs* transcript continue to increase across the course of infection to a level higher than that of the control, and this was associated with enhanced survival and lack of pathogen growth, traits indicative of increased resistance. Given its impact on suppressing the level of the *Drs* transcript, it may be that miR-193 acts as a broad limiter after an inflammatory response in a manner analogous to miR-155 and signaling through TLR4 (Schulte *et al.* 2013). The *miR-972-973-974* mutant has a complex phenotype, in that (following both PBS injection and infection) it exhibits enhanced survival, pathogen number, and a level of *Drs* above that of the control across the course of infection, and even at the point of injection. It may be that members of this cluster regulate Toll homeostasis and coordinate its response to infection. Involvement in Toll homeostasis is akin to the role of mir-8, which has been shown to suppress Toll signaling in adult fat body tissue under steady-state conditions, potentially by blocking the production of Toll (Choi and Hyun 2012; Lee and Hyun 2014). Regarding response to infection, miR-972 is predicted to target 18 genes from the Hippo signaling pathway, which is involved in regulating the level of *cactus* mRNA and promoting *Drs* production and survival in response to *S. aureus* infection (Liu *et al.* 2016).

On a final note, all the susceptible mutants exhibited an increase in pathogen number despite an elevated level of *Drs* transcript (except *miR-276b* regarding *Drs*, which was equivalent to the control). For the *miR-277-34* mutant, this may be explained by a confounding interaction between miR-277 and miR-34, but for *miR-317* and *miR-276b* only a single miRNA is deleted. One aspect to consider when interpreting miRNA data is that changes to even a single miRNA can affect the levels of thousands of proteins, often in a complementary manner (Selbach *et al.* 2008). This phenomenon explains how a single miRNA can affect a plethora of biological functions. For example, mir-8 has been shown to modulate the activity of the EGFR, Notch, and Toll pathways, act as a link between ecdysone and body growth, and affect motor neuron innervations of muscle (Carthew *et al.* 2017). Therefore, the disassociation between pathogen number and Toll signaling may be a consequence of a global dysfunction, where pathways that work in combination with Toll to protect the host from systemic infection are disrupted. Alternatively, other pathways that work independently of Toll to maintain general homeostasis/health may be affected.

We show, within the susceptible *miR-277-34* mutant, that the total amount of BCAAs is reduced compared to the control, consistent with previous findings implicating miR-277 as a key factor in controlling the catabolism of BCAAs (Esslinger *et al.* 2013). After 24 hr following PBS injection or infection, the amount of BCAAs was further reduced to a nominal level, thereby precluding conclusions regarding the latter. A possible explanation for this is that the shift in temperature—from 25 to 30°—accelerates metabolism, thereby driving the catabolism of BCAAs, given that its breakdown products are substrates for the TCA cycle (2016). In control flies, systemic *C. albicans* infection contributes to acceleration of the breakdown of BCAAs. Therefore, it is possible that signaling through Toll regulates BCAA metabolism by exerting an inhibitory effect on miR-277, thereby stimulating metabolism to provide energy to fight the infection; we note that miR-277-5p continually declines following *S. aureus* inoculation, which would theoretically release the repression on BCAA catabolism during infection. Alternatively, it may be that releasing a brake on metabolism, through the removal of miR-277, impacts positively on the outcome of Toll signaling; it has been seen in both mammals and *Drosophila* that alterations to metabolism can modulate immune signaling, with the two processes being antagonistic. A simple test for the latter would be to reduce energy production through manipulating the TCA cycle in a *miR-277* mutant, which, if the model is correct, should decrease the output of Toll signaling. Consistent with the connection between BCAAs and immune function, evidence shows that when they are lacking in the human diet, many aspects of immunity are impaired and susceptibility to pathogens is enhanced (Zhang *et al.* 2017).

### Genomic approaches to decipher miRNA involvement in systemic infection

In an attempt to identify miRNAs responsive to systemic infection, we performed a time course experiment on flies infected with different pathogens or injected with PBS, quantifying the level of miRNAs from whole-body extracts via microarray analysis. Approximately 90% of fold changes for infected over PBS were >0.5 and <2, including for the statistically separated (*P* < 0.1) miRNAs. Although it is not unusual to see significant changes of this magnitude, for example, in cultured cells following infection with different pathogens (Jin *et al.* 2014; Siddle *et al.* 2015), changes greater in magnitude are not uncommon. For example, the NF-ĸB-dependent expression of *miR-155* (Ceppi *et al.* 2009), which participates in an incoherent feedforward loop to regulate TLR signaling in hematopoietic cells, increased >20-fold in response to a variety of immune stimuli (Ryu *et al.* 2006; O’Connell *et al.* 2007; Siddle *et al.* 2015). More pertinent, a small-RNA-sequencing study identified 12 mature miRNA forms that were differentially expressed [greater than twofold (log2)] following systemic *M. luteus* infection in *D. melanogaster*; overall, 32 miRNAs were shown to be downregulated and 61 upregulated, with the vast majority of changes occurring 24 hr following infection (Li *et al.* 2017b). Notably, miR-34-3p/miR-277-3p/miR-311-3p/miR-973-3p were included in the upregulated miRNAs, and miR-34-5p/miR-276b-5p/miR-277-5p/313-5p/miR-317-3p and miR-317-5p were included in those that were downregulated. Regarding our miRNA microarray data, in general, we did not find consistency between miRNAs whose profiles changed over time for a particular pathogen and those differentially expressed relative to PBS. Therefore, we speculate that a critical aspect in the response to infection for some miRNAs is to maintain a relatively constant slope of production over time. This seems reasonable considering the time series heat maps, where the majority of statistically separated miRNA profiles switch from upregulated to downregulated (based on *Z*-score, thus changes given relative to the associated data set), or vice versa, at the later time points. Another consideration is that PBS injection may have an inconsistent effect on miRNA profiles; as such, their production may be similar to that for infected flies, but they will not be defined over time. It would be useful to know how miRNA profiles change in untreated flies. An interesting aside is that the switching at later time points (or general direction of the slope over time) may be a priming mechanism for a rapid response to further infection, once production of immune-stimulated transcripts has returned to basal level. For example, miR-958-5p and miR-34-5p tend to slope downward and upward, respectively, over the course of infection. Regarding the action of miR-958, this would in theory lead to increased production of Toll and Dif protein, whereas for miR-34 this would increase signaling through the Toll pathway.

The majority of mature miRNA forms (297 of 425) were not detected above background across the time course for PBS injected or infected flies, including both arms for members of the *miR-972-973-974* and *miR-959-964* clusters. This is notable given that deleting the former, or four members of the latter (Vodala *et al.* 2012), alters the response to systemic infection. More specifically, members of the *miR-959-964* cluster were shown to cycle – albeit at a relatively low abundance – via circadian transcriptional control, where expression was most prominent in the adult head fat body. Significantly, when this cluster was partially deleted, the time during the light:dark cycle at which flies were most susceptible to gram-negative infection was shifted (Vodala *et al.* 2012). That we did not observe enhanced susceptibility of this mutant to *C. albicans* may be partly explained therefore, by the timing of our infections; however, this phenomenon is likely to be a rare event, since most miRNAs do not exhibit circadian oscillations (Vodala *et al.* 2012). These observations bring to attention the dynamism and specificity of miRNA expression, and that having mature miRNAs at low abundance does not preclude important functional output. Considering this, using quantifying techniques with greater sensitivity (for example Small-RNA-Sequencing) and analyzing particular tissues or cells from infected flies, is likely to be more revealing. More so, if immune deficient flies are analyzed in parallel. Reporter constructs with rapid-turnover fluorescent proteins would also be a useful tool in the study of miRNAs. Therefore, in relation to immunity, we have provided a genome-wide map of potential NF-κB-binding sites that reside in close proximity to miRNA genes. However, we should be cautious when relating strength of those potential sites and role in immunity as there was no such correlation in our findings.

### Conclusions

We have identified six miRNA allelic mutant backgrounds that affect survival in response to systemic infection, the ability to control pathogen number, and production of Toll pathway-dependent transcripts. Furthermore, we reveal infection contributes to promoting BCAA catabolism, and through the susceptible *miR-277-34* mutant, identify a potential connection between BCAA catabolism and Toll signaling. Future studies carefully detailing the expression, tissue-specific requirement, and functional interactions between these miRNAs and their targets, will undoubtedly reveal new insight into the mechanisms required to cope with systemic infection.

## Supplementary Material

Supplemental material is available online at www.genetics.org/lookup/suppl/doi:10.1534/genetics.116.196584/-/DC1.
