# Supplementary figures and images for: MicroRNAs That Contribute to Coordinating the Immune Response in Drosophila melanogaster
Source: Genetics. 2017 Jul 13;207(1):163–78. doi: 10.1534/genetics.116.196584 (PMC5586370; doi:10.1534/genetics.116.196584)

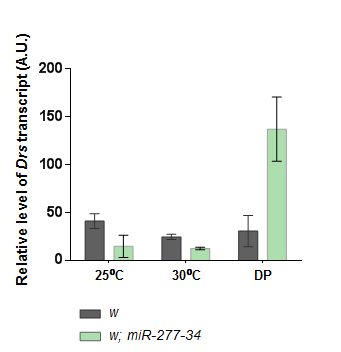

Supplement: Supplementary file 1 [file 163FigureS1.tif]

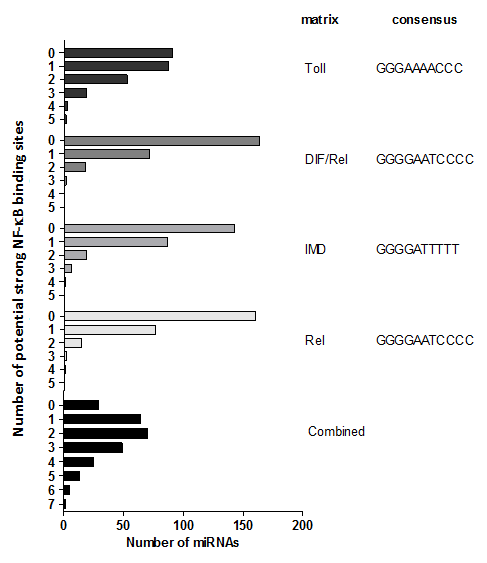

Supplement: Supplementary file 2 [file 163FigureS2.tif]

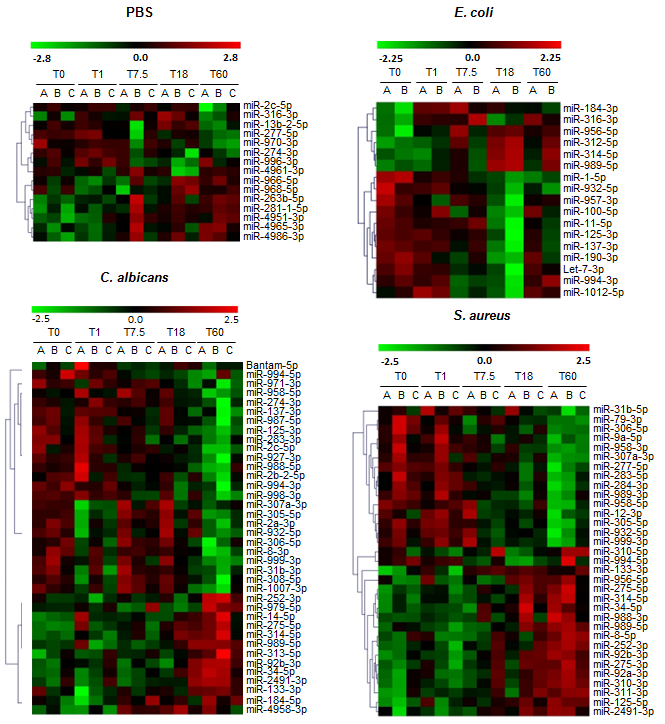

Supplement: Supplementary file 3 [file 163FigureS3.tif]

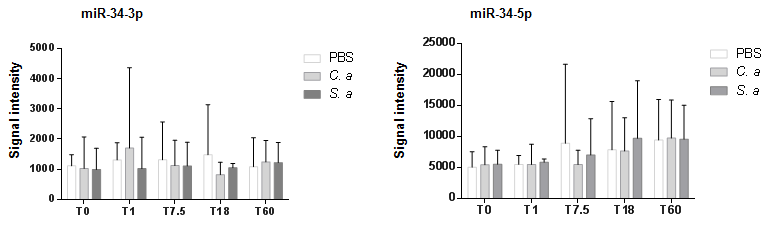

Supplement: Supplementary file 4 [file 163FigureS4.tif]
